# Supplementary material for: Patterns of Intron Gain and Loss in Fungi
Source: PLoS Biol. 2004 Nov 30;2(12):e422. doi: 10.1371/journal.pbio.0020422 (PMC532390; doi:10.1371/journal.pbio.0020422)
Supplement: Table S1 — Also available at http://genes.mit.edu/NielsenEtAl/. (4.3 MB ZIP). [file pbio.0020422.st001.zip › NielsenEtAl/html/986.html]

AN1296.1.NCU04410.1.MG05169.1.FG11594.1


```
 CLUSTAL W (1.82) Multiple Sequence Alignments - Introns Inserted


Sequence 1: NCU04410.1	840 aa
Sequence 2: FG11594.1	797 aa
Sequence 3: MG05169.1	831 aa
Sequence 4: AN1296.1	810 aa
Alignment Length: 879 aa
Number Identitical Residues: 299 aa
Alignment Score (without introns) 16289


MG05169.1 	---MATDAQGPGSLGNKAAASDG--MPNMVMPYEPQDPAVIAEMVRVLDEHTKGGAKGRF
NCU04410.1	MDEPLTDLRGPLAAKYTASTDYSKIADKFEVPHVAQDVHEVATLINGLENG--AKKKTSF
FG11594.1 	----------------------------MSAPYTPQDVQAVAAVVRALDNARKDKRKNGF
AN1296.1  	----------------------------------------MAKALEGARLENKGAKKKTF
          	                                        :*  :.      .   *  *

MG05169.1 	RIKKTKFAVTGSPSKVTVDSWKLQDWDYKKPGLPTYARGLFTTRLPNNVPEIAVRGYDKF
NCU04410.1	KVKKTTYEVAQSRDHIRVDSWRFHDWDYKKHDLPTYARGLFTTRHKDGTPEITTRGYDKF
FG11594.1 	SVKKTSFDVKGSADGIQVESWRMQDWDYKRPNLPTYARGLFTTRTRRNEPEIAVRGYDKF
AN1296.1  	NCRKSTFKVG-KAGDISADSWKFMDWDYKRRDLPTYARGLFTAKRKDGTTEIVTRGYDKF
          	  :*:.: *  . . : .:**:: *****: .**********::   . .**..******

MG05169.1 	FNVGEVPETNWENIIKNSKGPYELSLKENGCIIFISGLHDDTLLVCSKHSTGARGDIEIS
NCU04410.1	FNVDEVTETKWENIRNRTRGPYELTLKENGCIIFVSGLADDTLLVCSKHSTGDRSDVETS
FG11594.1 	FNVEEVPETKWEKIFAQTQGPYELTLKENGCIIFIAGLEDDTLVVCSKHSTGDREDIQVS
AN1296.1  	FNVDEVSATQWRNIEAYTEGPYELSVKENGCIIFISGLEDGSLLVCSKHSTGARADTDRS
          	*** **. *:*.:*   :.*****::********::** *.:*:******** * * : *

MG05169.1 	HANAGEKALDKQLAKIGKTRADLARELRERNATAIAELCDDSFEEHILAYGPDKAGLYLH
NCU04410.1	HASAGERRLEEQLARIGKTKADLARELRRRNVTAVAELCDDSFEEHILAYGPDKAGLYLH
FG11594.1 	HASAGEQRLEQQLAAVGKTKADLARELRKRNVTAVAELCDDEFEEHILEYGPDKAGLYLH
AN1296.1  	HAQVGEEWIERHVATVGKSTAELAKQLREMNATAVGELCDDSFEEHVLAYDSAAAGIYLH
          	**..**. ::.::* :**: *:**::**. *.**:.*****.****:* *..  **:***

MG05169.1 	GINVNQPYFMTYPGPAVNSFADDWGFKQTGLLIMDNIKEVKTFLEDVAETGAYGGRDVEG
NCU04410.1	GMNLNLPEFMTYPSPVVQKFAEEWGFIKTGLIVIDTIDEVKAFLEEVAESGAHDGRDVEG
FG11594.1 	GMNLNLPQFATYPSRYVQEFADEWAFRKTGLMVMDDIHQVKSFLEEVAETGAHDGRDVEG
AN1296.1  	GINYNLPEFATLPSSEVHRFADAWGFKKANFVVYDDLSTVKKFLEKCAETGSWDGRDTEG
          	*:* * * * * *.  *: **: *.* ::.::: * :  ** ***. **:*: .***.**

MG05169.1 	FVIRCKLKDRPNTDPGTYSDWFFKYKFEEPYLMYRQWRECTKALIAGHAPKIRKHKKITE
NCU04410.1	FVIRCKMSYQPGQQP--FQDWFFKYKFEEPYLMYRQWRECTKAIIAGKPPKFKKHVKITE
FG11594.1 	FVIRCKMSQDPATQP--FQDWFFKYKFEEPYLMYRQWRECTKALIAGKQPKFKKHTKITE
AN1296.1  	FVIRCKMSEN-GHGP--YRDWFFKYKFEEPYLMYRQWRECTKAILSGKAPKIKKHKKITE
          	******:.      *  : ************************:::*: **::** ****

MG05169.1 	EYLYYAKTRLAEDSSLAKAYNQNHGIIALRDDFLKYKNMKGSDAANLDRDVATNEPEPDN
NCU04410.1	EYLLYARQRLAGDRELAKRYNQNHGIIALRNDFLAFKNLKGSDAANFE---ALHGGGAAA
FG11594.1 	EYLLYARRRLVADPKLGKEYNSNHGIIALRNDFLTFKNLKGADAANLG---DLDCP-AMT
AN1296.1  	EYLAYARQQFALHPKLREQYQHNHGIISMRDGFLKERGLNGAQIIQME----------AE
          	*** **: ::. . .* : *: *****::*:.**  :.::*::  ::             

MG05169.1 	SVVGNVILVPIATIGCGKTTIAVALRTLFPEAFGHFQNDSITQKKGRPAIFTRNVLEQLK
NCU04410.1	DVESDIILVPIATIGCGKTTVGLALTHLF--GWGHVQNDNITGK-ARPPRFTKALLDILD
FG11594.1 	EVTRDVILCPIATIGCGKTTIAMGLSHLF--GWGHVQNDNISGK-GRPPRFTKMVLDELK
AN1296.1  	NVTRDVVLVPIASIGCGKTTVGLALTKLF--GWGQVQNDNIPKQKNKPKQFVANILNLLM
          	.*  :::* ***:*******:.:.*  **  .:*:.***.*. :. :*  *.  :*: * 

MG05169.1 	T-KRVVIADRNNAWRRERTQILGDVKNMLPAAKLVALHFDHD----NSMEVRKLTYERVF
NCU04410.1	EGVPAVYADRNNAQKHERKQLITDVKIQHARARLVALHFVHN----DLEKVREVCRDRVF
FG11594.1 	D-HPAVVADRNNAQRHERKQIITDVKLQHSTAKLVCLNFKHDEE--AIDEIRRITQERIV
AN1296.1  	D-KPAVIADRNNHQRRERSQLINDIVAGSPNARFVALHYVHEPKDMMLPLIREVTRKRVL
          	    .* *****  ::**.*:: *:    . *::*.*:: *: ..     :*.:  .*:.

MG05169.1 	QRGDNHQNIKVQELGKAKVMDIMNGFLDGLEPYSAFSKPDADFSAAIGLNPLAGSRTNLE
NCU04410.1	ARGDNHQTIQAASD-MNKVRGIMEGFINRFEPCDPEQKPDSEFDDIIDLDPSAGSRQNLE
FG11594.1 	TRGDNHQTIHAASD-KDKFIGVMEGFIKRFEPCNPHGRPDDGFDAFIDLDPTAGSRQNLE
AN1296.1  	DRGDNHQTIRAGTKDTDEVLGIMEGFLRRFETVDVDHEPDQNFDEVIDLDVTASSRENLE
          	 ******.*:.   .  :. .:*:**:  :*. .   .**  *.  *.*:  *.** ***

MG05169.1 	VVISKLHQFYPNLLPRVPTGEEMDKAIDTALA-YDPKLEQLAVLPAAA------NNN---
NCU04410.1	TVIKELHRMYPKFMPNIPSAAEMDEAIQYALEGYKPDLRHVIPDRSNK------NKNQPK
FG11594.1 	VVVTQLHKLFPNLVGEIPSSGTLDAAIDCALG-YKPEFRHDIPDRGKK------NSQQQK
AN1296.1  	KVVNALHSAYPQLVTKVPSAQELDDAINYATSEYSVEVDHSASYGQSKPSKQSNKSNSNK
          	 *:. **  :*::: .:*:.  :* **: *   *. .. :        .:..:.:.:. .

MG05169.1 	-----------KGGIEYMSIDIPSQAVLAALEDAFKD--ASPEVGRMYGYLKQQRRIQPA
NCU04410.1	QNGQQQQQKQKKRPLEYMSVDMTTKDVLNTLEKAFAA--AGPEQSRFFKQLQGTRRVQPK
FG11594.1 	Q----QVKTPKPRKMEYMSVSIPTQDVNSALDNAFRN--VPASTSQLYTQLKQTRRVQPK
AN1296.1  	NGGSEINIEAKVRKIEYFGISLPTSSVTDLLHSLFANPAITPEKARLYHHLVNSRRVQPA
          	.....     .   :**:.:.:.:. *   *.. *  .:  .. .:::  *   **:** 

MG05169.1 	FHVTLMHKSGAKTSPENQELWTRYTQTLKVAHDDAKSKGIVFRAAETPSLGECDVQLER~
NCU04410.1	FHVTLMHRAGAKENPQ---LWDRYLRIN-ELEGPNSPDG---------KLGEIDVQLER~
FG11594.1 	FHVTLLHKAASVNHPE---LWEQYTALHKEVEAAGNPEG---------KVGECDVMLER0
AN1296.1  	FHVTLIHKALKTAHPD---VWEDLVNRYVGQMKKNPPKD----HALTPPLGSARVRLER~
          	*****:*::     *:   :*               ...     : :. :*.  * *** 

MG05169.1 	VVFDDRLMAIVVRIIDK--------------DDKWKCINRVPHITIGTLDNTVPPKESND
NCU04410.1	VVYDDRIMAVVVRLIPRPGDEHANPDSSEPAEPKWQCTNRVAHITVGTRDDSVKPRESND
FG11594.1 	VVFDDRIMAIVVRLADQ--------------DDRWQCVNRVAHITVGTRDNTVKPKESND
AN1296.1  	LIWDDKIMTFVARIMPG---EPENSGDNAEPQPDWICVNPLPHVTVGTVSPAVKPKESND
          	:::**::*:.*.*:      .  .....  .:  * * * :.*:*:** . :* *:****

MG05169.1 	MLALWIDGQQEARRGTIRETGIEGRPIVKGIVKAVLSKF-
NCU04410.1	LLVKWLEHGTGAETG-IYDLLIDGKPTLQGVVRGVLSR--
FG11594.1 	LLARWLEVGSSPETK-IGEVVFAGRPTVKGTVMPVLSRF-
AN1296.1  	LLQRWLQEGSQ-GTG-IWEMEIPGVKVVNGTVNVTMSRGK
          	:*  *::         * :  : *   ::* *  .:*: .
```
